# Prediction model for daily feed intake during the growing period in floor-reared *Wanxi White Geese* based on machine learning and feeding behavior features

**DOI:** 10.1016/j.psj.2026.106991

**Published:** 2026-04-23

**Authors:** Deqin Xiao, Weichao Lin, Youfu Liu, Shaohai Peng, Dakang Guo, Kejian Liu

**Affiliations:** a College of Mathematics Informatics, South China Agricultural University, Guangzhou, 510642, China; b Key Laboratory of Smart Agricultural Technology in Tropical South China, Ministry of Agriculture and Rural Affairs, Guangzhou, 510642, China; c Guangdong Laboratory for Lingnan Modern Agriculture, Guangzhou, 510642, China

**Keywords:** Radio frequency identification, Electronic feeder, Feature engineering, Stage-wise modeling, Feed conversion ratio

## Abstract

To improve the efficiency of obtaining daily feed intake (DFI) of *Wanxi White geese* under floor-rearing conditions, this study used 200 *Wanxi White geese* as experimental animals. Individual information such as body weight (BW) and feeding behavior data were collected using Radio Frequency Identification (RFID) technology and an electronic feeder system, and feature indicators were constructed on a daily basis to form four feature sets. Four machine learning (ML) algorithms were used to develop 16 DFI prediction models by combining different feature sets, and model performance was evaluated across three weekly age segments during the growing period (22–28 d, 29–35 d, and 36–42 d). The results showed that ensemble learning models generally outperformed the linear model, and the integrated feature set combining individual information and feeding behavior features exhibited the most stable performance. The RF–IF_FB model performed best in the 22–28 d stage (R² = 0.75, RMSE = 13.96 g), the GBR–IF_FB model performed best in the 29–35 d stage (R² = 0.77, RMSE = 14.65 g), and the XGB–IF_FB model performed best in the 36–42 d stage (R² = 0.77, RMSE = 14.82 g). Furthermore, weekly feed conversion ratio (FCR) was estimated based on predicted DFI, and geese were classified according to the mean and standard deviation of FCR. The predicted grades showed high agreement with the measured grades (kappa = 0.74–0.80). These findings indicate that ML and feeding behavior features can be used to predict DFI of *Wanxi White geese* under floor-rearing conditions and support efficiency grading based on FCR derived from predicted values.

## Introduction

Global goose meat production has increased markedly, rising from approximately 150,000 tons in 1961 to a projected 4.75 million tons by 2027. During 2023–2027 alone, production is expected to increase by 246% compared with the historical average (Dumlu, 2024), indicating that the goose industry is entering a period of rapid expansion. The main products of the goose industry include goose meat, foie gras, and goose down (Kozák, 2021). China plays a dominant role in the global goose industry, accounting for 95% of world goose meat production (Akhtar et al., 2020). As China becomes increasingly integrated into global poultry trade and supply chains, international requirements for product quality consistency, food safety, and traceability—often linked to export market access—have continued to tighten. These pressures have accelerated the transition of China’s poultry production from extensive farming toward precision-oriented and intelligent management (Zhang et al., 2025).

**Feed conversion ratio (FCR)** is a key indicator for evaluating production efficiency in poultry farming, reflecting the amount of feed required per unit of body weight gain. A lower FCR indicates higher feed utilization efficiency, thereby directly affecting production costs and economic returns (Prakash et al., 2020). With fluctuations in the prices of major feed ingredients such as corn and soybean meal, feed costs in poultry production have accounted for more than 60% of total production costs (Donohue and Cunningham, 2009; Willems et al., 2013). Therefore, scientifically determining and optimizing FCR to improve growth performance and economic benefits has become a major focus of the industry.

The calculation of FCR relies on the acquisition of individual-level **feed intake (FI)** and **body weight (BW)** data. Floor rearing remains the predominant production system for geese (Tang et al., 2023). Under floor-rearing conditions, geese move freely and are spatially dispersed, which complicates individual identification. In addition, feeding sites are typically non-centralized, making it difficult to obtain continuous and accurate measurements of FI at the individual level. These constraints limit the implementation of refined feed efficiency management and the effective identification of high- and low-performing individuals.

In recent years, the development of **Radio Frequency Identification (RFID)** technology and the Internet of Things has provided new approaches for individual identification and automated data collection under group-housing conditions. Automated systems have been developed to measure FI in ruminants such as cattle and sheep (Oliveira et al., 2018; Muir et al., 2020), and studies have also attempted to achieve automated collection of BW- and FI-related data in poultry (Tu et al., 2011; Peng et al., 2022). However, these poultry FI measurement systems often rely on precise feed weighing and complex hardware integration, resulting in high equipment and maintenance costs, which limits their application in commercial production. In contrast, feeder visiting behavior (e.g., time spent at feeders and visit frequency) and BW are easier to obtain and can be continuously recorded using multiple sensing solutions. Therefore, exploring methods to predict FI based on accessible growth and behavioral traits may reduce dependence on costly precise feed-weighing modules while providing a feasible pathway for evaluating individual feed efficiency.

Meanwhile, with the rapid development of big data and artificial intelligence, some studies have attempted to estimate broiler FI using non-contact approaches such as sound or image analysis (Aydin et al., 2014; Meng et al., 2024). However, these approaches are susceptible to interference from noise, occlusion, and changes in lighting conditions, and their stability under group-housing environments remains to be improved. Thus, developing reliable FI prediction models using more accessible and stable multi-source information has become an important research direction. In this context, **machine learning (ML)** algorithms have gained increasing attention in livestock and poultry production performance prediction because they can capture complex nonlinear relationships between multidimensional features and target traits. Previous studies have used ML algorithms to predict body weight in growing pigs based on feeding behavior data and divided continuous growth data into fixed-length time windows for model training and validation (He et al., 2021), or developed FI prediction models for dairy cows based on feeding time, rumination time, and diet composition, while identifying the key features contributing to model performance (Shangru et al., 2022). In addition, studies have derived FCR based on predicted FI in beef cattle and classified animals according to the mean and standard deviation of FCR to grade growth performance (Davison et al., 2023). However, for poultry, particularly geese, systematic research and quantitative evaluation are still lacking regarding how to develop FI prediction models under floor-rearing conditions using accessible individual growth traits and feeder visiting behavior data, and how to further validate the feasibility and stability of FCR-based efficiency grading.

Based on this background, the objectives of this study were as follows:

(1). Under floor-rearing conditions, to develop ML-based prediction models for **daily feed intake (DFI)** using individual BW and feeder visiting behavior data collected with RFID leg bands and an electronic feeder system;
(2). To compare the prediction performance and stability of different ML algorithms and feature set combinations across different weekly age stages (22–28 d, 29–35 d, and 36–42 d), and to evaluate the applicability of the models throughout the growing period;
(3). To estimate weekly FCR based on predicted DFI and to validate the reliability of FCR derived from predicted values for individual feed efficiency evaluation and performance grouping.

## Materials and methods

### Experimental animals and experimental setting

**Fig. 1:**
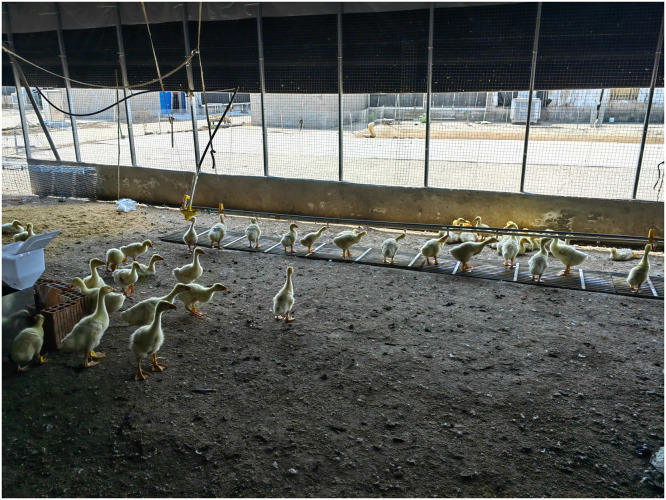
Layout of the experimental site for floor-reared *Wanxi White geese.*

The data in this experiment were obtained from a breeding farm located in Jin’an District, Lu’an City, Anhui Province, China, and the trial was conducted from August 1 to October 1, 2025. The experimental animals were *Wanxi White geese*, an excellent local medium-sized goose breed native to Lu’an City and surrounding areas, characterized by broad adaptability, strong feeding capacity, tolerance to cold and heat, and tolerance to roughage, with good flocking behavior and ease of management under floor-rearing conditions. The experimental site was arranged around a shed (Fig. 1), and the flock could move freely inside the shed or in the area surrounding the shed. A total of 200 healthy *Wanxi White geese* were selected for the experiment, with an approximate sex ratio of 1:1. The geese entered the experimental site at approximately 15 d of age for acclimation and training, and data were formally collected from approximately 22 d of age (3 wk of age) to 42 d of age (6 wk of age), for a total of 3 wk. This age range corresponds to the growing period, during which geese grow rapidly, which is conducive to observing the effects of individual characteristics and feeding behavior on FI. During the experiment, all geese were fed the same diet to ensure consistent nutrient supply and to reduce the confounding effects of dietary differences on FI, thereby enabling a more accurate evaluation of the effects of individual growth traits and feeding behavior on FI. The diet was formulated based on a commonly used local fattening diet for meat geese and consisted of corn (63%), wheat bran (10.5%), soybean meal (14%), oil (2.5%), and other trace elements (5%), which met the nutritional requirements for growth at this stage.

### Data collection and processing

#### Individual identification and collection of identity information

**Fig. 2:**
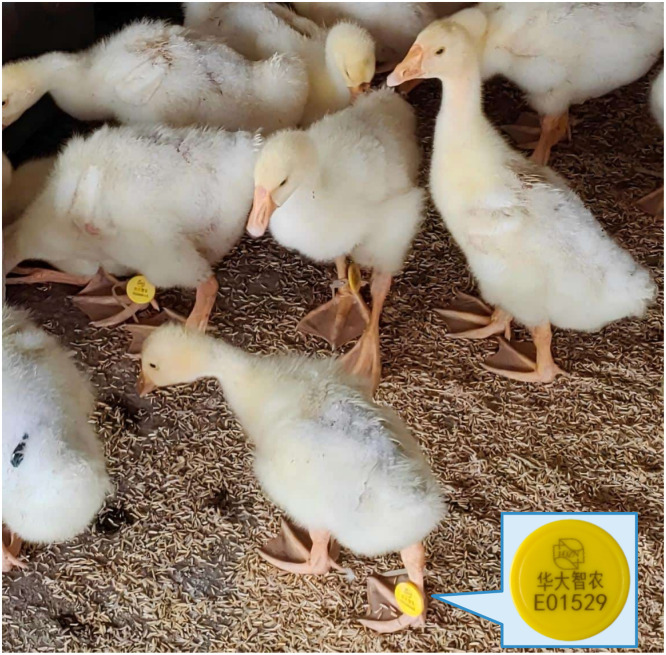
RFID leg band used for individual identification of *Wanxi White geese*.

In this study, customized RFID leg bands (Guangzhou SCAU Smart Agriculture Technology Co., Ltd., Guangzhou, China) were used for individual identification of each goose (Fig. 2). Compared with traditional wing tags that require puncturing the wing, RFID leg bands do not cause tissue damage to poultry, which can effectively reduce animal stress responses and improve welfare. Previous studies have reported adverse effects of wing tags on birds (Kinkel, 1989; Trefry et al., 2013; Curk et al., 2021). The leg band casing used in this study was made of ABS engineering plastic, with an RFID chip embedded inside containing a unique identification code, and the weight was approximately 3 g. The leg band was worn on the left or right leg of the goose and the tightness could be freely adjusted according to the leg size, ensuring stable identification without interfering with normal activities of the geese.

#### Collection of feeding behavior and body weight data

**Fig. 3:**
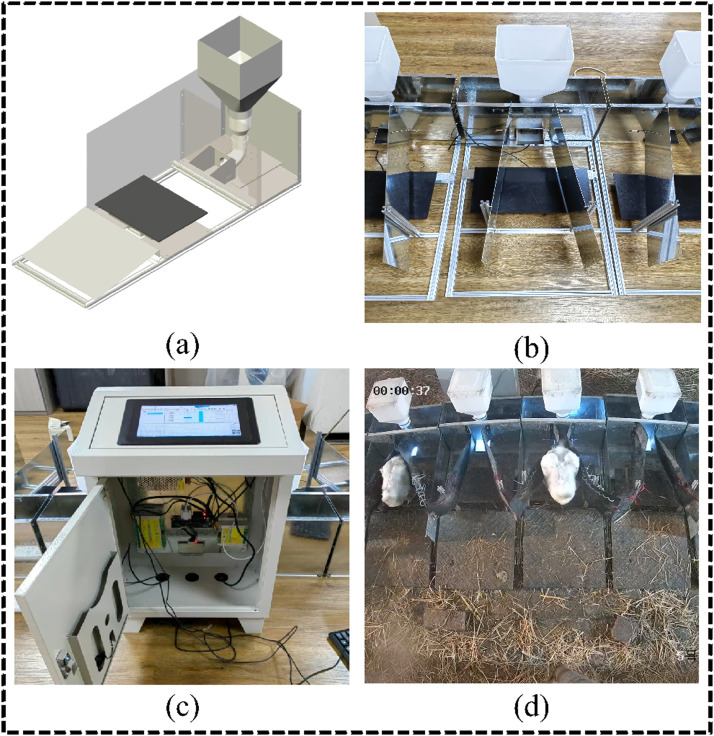
Electronic feeder system used to record feeding behavior and body weight of *Wanxi White geese*, (a) Three-dimensional schematic of the electronic feeder, (b) Photograph of the feeder installed in the experimental facility, (c) The computer that controls the feeding system, (d) Observe the feeding situation through surveillance video.

An electronic feeder system (Guangzhou SCAU Smart Agriculture Technology Co., Ltd., Guangzhou, China) was installed in the experimental goose house. As shown in Fig. 3, two movable limiting rails were installed at the entrance of the feeder, and the passage width could be flexibly adjusted according to changes in goose body size at different ages, ensuring that only one goose was allowed to enter the feeding area at any time, thereby avoiding confusion of individual feed intake data. The feeder integrated an RFID antenna and a high-precision pressure sensor to read the ID from the leg band tag in real time and to obtain BW and **each feed intake (EFI)**, while also recording the **time spent at the feeders (TSF)** and the **number of visited feeders (NVF)**. Each feeder could serve 20 geese. A total of 10 feeders and one water line were deployed in the center of the experimental site, and all feeders were controlled by the same computer. Feeders and water were continuously available, simulating ad libitum feeding. A surveillance camera was installed above the feeders to observe the feeding behavior of the geese.

Although the electronic feeder used in this experiment integrated a weighing module that could directly obtain EFI and calculate the true DFI, this module was mainly used to provide reference values for model training and validation. This study aimed to verify the feasibility of predicting DFI based on RFID identification and feeder visiting behavior features (e.g., time spent and visit frequency), so that low-cost and scalable estimation of individual feed intake and feed efficiency evaluation can be achieved in future floor-rearing production scenarios without weighing modules.

#### Data preprocessing

To ensure the accuracy and robustness of the training data, the raw records were subjected to data preprocessing and quality control before model development. The raw dataset mainly comprised individual RFID identification information, EFI, TSF, NVF and BW.

First, data completeness was checked and records with failed RFID identification or missing key information were removed. For a small number of missing values occurring at consecutive time points, interpolation using adjacent time points was applied to restore continuity without compromising the integrity of the time series. To exclude abnormal events arising from non-feeding visits, brief stays, or false triggers during a single feeding event, records with TSF < 5 s and EFI < 1 g were discarded. For BW, if the BW value at a given time point exhibited abrupt fluctuations relative to adjacent time points that were inconsistent with biological growth patterns (e.g., a marked short-term decrease), the record was considered an outlier and was either removed or corrected using linear interpolation between neighboring time points.

Because single feeding events have strong randomness, data were aggregated on a daily basis to improve model stability. For each goose, daily feeding behavior and BW information were calculated as follows: DFI was the sum of all EFI values for each goose on that day; **daily number of visited feeders (DNVF)** was the total number of NVF for each goose on that day; **daily time spent at the feeders (DTSF)** was the sum of all TSF values for each goose on that day; **daily body weight (DBW)** was the mean of all normal BW records for each goose on that day; and **daily average visit duration (DAVD)** was calculated as the ratio of DTSF to DNVF for each goose on that day.

### Development of the feed intake prediction models

Goose DFI is not only related to individual growth traits but is also closely associated with feeding behavior. Based on this, this study used 200 *Wanxi White geese* (22–42 d of age) as experimental animals. Four commonly used ML algorithms and four feature sets were selected to develop 16 DFI prediction models to compare the predictive ability of different algorithm–feature combinations and to identify the model with the best overall performance for subsequent analyses.

#### Construction of feature sets

In this study, four feature sets were constructed for model comparison, including:

(1). **Individual-level features (IF)**, including sex, age, and DBW, which were used to characterize basic individual attributes and growth status of the geese;
(2). **Feeding behavior features (FB)**, including DTSF, DNVF, and DAVD, which were used to reflect behavioral patterns of the geese at the feeders;
(3). **The age + sex + feeding behavior feature set (Age_Sex_FB)**, which added age and sex information to FB (excluding DBW), to evaluate the contribution of basic individual features and behavioral features to DFI prediction in the absence of BW information;
(4). **The combined feature set (IF_FB)**, which integrated all features in IF and FB to evaluate the improvement in DFI prediction performance achieved by multi-source information fusion. The specific composition of each feature set is shown in Table 1.

**Table 1:** Composition of feature sets used for daily feed intake prediction models.

Feature set	Number of features	Age	Sex	DBW	DTSF	DNVF	DAVD
IF	3	√	√	√			
FB	3				√	√	√
AS_FB	5	√	√		√	√	√
IF_FB	6	√	√	√	√	√	√

DBW: daily body weight; DTSF: daily time spent at the feeders; DNVF: daily number of visited feeders; DAVD: daily average visit duration.

#### Selection of machine learning algorithms

The four commonly used ML algorithms selected in this study were least absolute shrinkage and selection operator **Least Absolute Shrinkage and Selection Operator (LASSO), eXtreme Gradient Boosting (XGB), Random Forest (RF)**, and **Gradient Boosting Regression (GBR)**. All ML algorithms were implemented using the Scikit-learn library in Python.

LASSO is a modeling approach that introduces L1 regularization into the linear regression framework. By applying a penalty term to the regression coefficients, LASSO shrinks parameters such that some coefficients approach zero, thereby achieving feature selection and model simplification (Tibshirani, 1996). This method is suitable for scenarios with a relatively large number of variables or multicollinearity, and it can reduce model complexity and alleviate overfitting to some extent while improving interpretability. In this study, the regularization strength parameter α of LASSO was tuned using cross-validation, with a candidate range of α ∈ {0.001, 0.01, 0.1, 1, 10}. The value that achieved the minimum mean squared error on the training set was selected.

XGB is an efficient implementation of gradient-boosted decision trees. Based on the idea of additive modeling, it iteratively learns residuals and continuously optimizes the objective function to improve overall fitting performance (Chen and Guestrin, 2016). XGB can effectively capture nonlinear relationships and higher-order interaction effects, and it controls complexity through regularization terms and pruning strategies, typically providing high accuracy and strong generalization ability for complex feature structures and nonlinear regression problems. In this study, the main hyperparameters of XGB included the learning rate (learning_rate), maximum tree depth (max_depth), subsample ratio (subsample), and feature sampling ratio (colsample_bytree). The candidate parameter ranges were set as follows: learning_rate ∈ {0.05, 0.1}, max_depth ∈ {3, 4, 5}, subsample ∈ {0.8, 1.0}, and colsample_bytree ∈ {0.8, 1.0}.

RF is an ensemble learning algorithm based on the bagging strategy. It generates multiple bootstrap samples from the training data and randomly selects a subset of features at each node split to train multiple independent decision trees and aggregate their outputs (Breiman, 2001). This method is robust and insensitive to noise and outliers, and it effectively reduces variance risk in single models, making it suitable for regression tasks with complex data structures. In addition, RF provides feature importance scores to facilitate interpretation. In this study, the main hyperparameters of RF included the number of trees (n_estimators), maximum tree depth (max_depth), and the minimum number of samples per leaf node (min_samples_leaf). Hyperparameter tuning was performed using grid search, with candidate ranges of n_estimators ∈ {200, 500}, max_depth ∈ {None, 10, 20}, and min_samples_leaf ∈ {1, 3, 5}.

GBR is also an ensemble regression method within the boosting framework. Its core idea is to sequentially add multiple weak learners (typically shallow regression trees) and progressively reduce overall error by fitting residuals (Friedman, 2001). GBR performs well in learning complex patterns and can achieve favorable performance in the bias–variance trade-off; however, its performance is sensitive to parameter settings. Therefore, cross-validation is typically required to determine appropriate learning rates, tree depths, and the number of iterations. In this study, the learning rate (learning_rate), number of weak learners (n_estimators), and maximum tree depth (max_depth) of GBR were tuned, with the search ranges set as follows: learning_rate ∈ {0.01, 0.05, 0.1}, n_estimators ∈ {200, 500}, and max_depth ∈ {2, 3, 4}.

#### Data splitting and model evaluation strategy

The training and test sets were split at the individual level, with 70% of the geese randomly selected as the training set and the remaining 30% used as an independent test set. During model training, hyperparameter tuning was performed only within the training set, and 10-fold cross-validation was used to evaluate the predictive performance of different parameter combinations. To improve comparability among variables with different scales and enhance the stability of model training, all algorithms standardized the input variables before model development. Models were then retrained on the full training set using the optimal hyperparameters and evaluated on the independent test set to assess generalization performance.

In addition, to compare the stability of model performance across different growth stages, the continuous 21-d daily data from 22 to 42 d of age were divided into three 1-wk time segments (22–28 d, 29–35 d, and 36–42 d), and the prediction performance of each model was evaluated for each weekly stage. Through a comprehensive comparison of the 16 models across different time windows, the model with the best overall prediction accuracy and stability was selected for subsequent FCR analysis based on predicted DFI.

#### Grading based on feed conversion ratio

To further evaluate the application value of the DFI prediction models in production management, FCR was calculated based on the predicted DFI values, and geese were graded accordingly to assess growth performance. To reduce the influence of daily fluctuations on the results, FCR was calculated on a weekly basis, and the formula was as follows:

(1) $$FCR=\frac{WFI}{WWG}$$

where WWG represents weekly weight gain and WFI represents weekly feed intake. WFI was calculated based on the observed and predicted DFI values, thereby obtaining the observed and predicted values of FCR.

In this study, a threshold-based grading method was used to classify goose growth performance based on FCR. Using the overall mean and standard deviation of FCR across all individuals, geese were classified into three categories: the high-efficiency group (FCR ≤ mean − SD), the medium-efficiency group (mean − SD < FCR < mean + SD), and the low-efficiency group (FCR ≥ mean + SD). The above grading was conducted based on both the observed and predicted FCR values, yielding measured grades and predicted grades. To evaluate the agreement between predicted and measured grades, Cohen’s kappa coefficient (K) was used to quantify the level of concordance between the two grading results after excluding agreement due to chance. Through threshold-based grading and K-based agreement evaluation, this study validated the feasibility of deriving FCR from predicted DFI and conducting efficiency grading, providing a methodological basis for individual feed efficiency selection and production management decision-making.

### Model evaluation metrics and experimental workflow

In this experiment, **mean absolute error (MAE), coefficient of determination (R²)**, and **root mean square error (RMSE)** were used to comprehensively evaluate the performance of the DFI prediction models.

MAE measures the average absolute magnitude of the deviation between predicted and true values and is one of the most commonly used error evaluation metrics in regression tasks. Based on absolute errors, this metric can intuitively reflect the average degree of deviation between predictions and actual observations; smaller values indicate that the overall predictions are closer to the true values. Compared with squared-error metrics, MAE is less sensitive to a small number of extreme errors and therefore provides more stable evaluation when the data contain outliers or exhibit a skewed distribution. The formula for MAE is as follows:

(2) $$MAE=\frac{1}{n}\sum_{i=1}^{n}\left|\hat{y_{i}}-y_{i}\right|$$

where n represents the number of samples, $y_{i}$ represents the observed value of the i-th sample, and $\hat{y_{i}}$ represents the predicted value of the i-th sample.

RMSE is used to measure the overall level of deviation between predicted and observed values. It is obtained by taking the square root of the mean of squared errors; therefore, it assigns greater weight to larger errors and can better reflect model stability and risk when large deviations occur. RMSE has the same unit as the original data, which facilitates intuitive interpretation. A smaller RMSE indicates lower overall prediction error and better model fit. The formula for RMSE is as follows:

(3) $$RMSE=\sqrt{\frac{1}{n}\sum_{i=1}^{n}{\left(y_{i}-\hat{y_{i}}\right)}^{2}}$$

where $y_{i}$ is the observed value of the i-th sample, $\hat{y_{i}}$ is the predicted value of the i-th sample, n is the number of samples, and ${\left(y_{i}-\hat{y_{i}}\right)}^{2}$ is the squared error of the i-th sample.

R² is used to evaluate the goodness of fit of a model to the observed data, reflecting the proportion of the variance in the observed values that can be explained by the model, that is, the explanatory power of the model for variation in the dependent variable. R² typically ranges from 0 to 1, with larger values indicating that the model more adequately captures data variation and provides a better fit; when R² approaches 1, the predicted values are more consistent with the observed values. The formula for R² is as follows:

(4) $$R²=1-\frac{\sum_{i=1}^{n}{\left(y_{i}-\hat{y_{i}}\right)}^{2}}{\sum_{i=1}^{n}{\left(y_{i}-\bar{y}\right)}^{2}}$$

where $y_{i}$ is the observed value of the i-th sample, $\bar{y}$ is the mean of the observed values, $\hat{y_{i}}$ is the predicted value of the i-th sample, and n is the number of samples.

The kappa coefficient was used to evaluate the agreement between growth performance grading results based on predicted and observed values. Compared with overall accuracy, the kappa coefficient can more objectively reflect the consistency between the two grading results after accounting for agreement due to chance. It ranges from −1 to 1, with larger values indicating higher agreement. The formula for the kappa coefficient is as follows:

(5) $$K=\frac{P_{o}-P_{e}}{1-P_{e}}$$

where $P_{o}$ is the observed agreement (i.e., the proportion of cases in which the predicted grade and the measured grade are exactly the same), and $P_{e}$ is the expected agreement by chance (i.e., the theoretical proportion of agreement that may occur under random grading conditions).

**Fig. 4:**
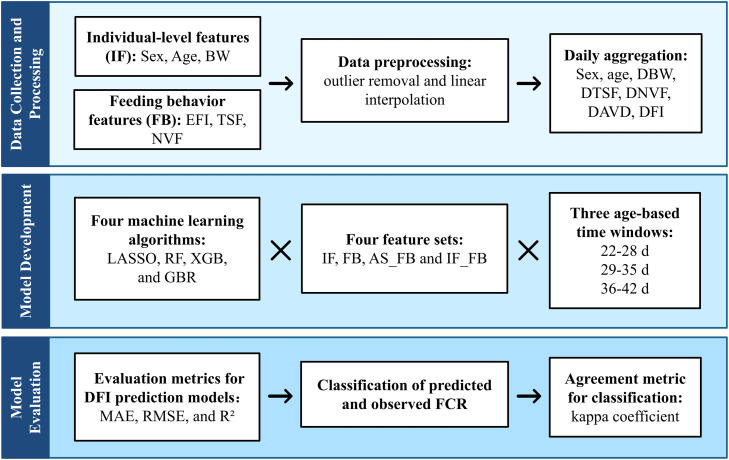
Experimental workflow of model development and evaluation. BW: body weight; DBW: daily body weight; NVF: number of visited feeders; DNVF: daily number of visited feeders; DFI: daily feed intake; EFI: each feed intake; TSF: time spent at the feeders; DTSF: daily time spent at the feeders; DAVD: daily average visit duration; FCR: feed conversion ratio; GBR: Gradient Boosting Regression; RF: Random Rorest; XGB: eXtreme Gradient Boosting; LASSO: Least Absolute Shrinkage and Selection Operator; AS_FB: age, sex, and feeding behavior features.

The experimental workflow of this study is illustrated in Fig. 4.

## Results

### Statistical analysis

To ensure the reliability of DFI and feeding behavior data, missing values were removed from the raw dataset, abnormal feeder visits were filtered, and continuity checks were performed, resulting in cleaned daily-scale data (Table 2). The results showed that the mean BW of geese increased continuously with age during the 22–42 d period, rising from 1245.5 g at 22 d to 2491.3 g at 42 d.

**Table 2:** Descriptive statistics of growth, feeding behavior traits, and feed efficiency of *Wanxi White geese* during the growing period.

Age (d)	Feature	Mean	SD	Minimum	Maximum
22	BW (g)	1245.5	224.2	1013.5	1526.2
28	BW (g)	1614.2	262.1	1354.9	1924.3
35	BW (g)	2023.4	303.5	1727.4	2375.5
42	BW (g)	2491.3	345.6	2126.3	2726.8
22-28	DNVF (times/d)	15.4	3.3	8	25
29-35	DNVF (times/d)	17.8	3.9	7	24
36-42	DNVF (times/d)	18.2	3.4	6	25
22-28	DFI (g/d)	133.2	20.6	78.2	185.5
29-35	DFI (g/d)	158.4	32.2	102.3	224.2
36-42	DFI (g/d)	187.5	44.8	126.6	239.5
22-28	DTSF (min/d)	32.7	6.2	10.2	52.5
29-35	DTSF (min/d)	35.4	7.0	9.4	49.6
36-42	DTSF (min/d)	36.3	7.8	11.9	50.4
22-28	DAVD (min/visit)	2.12	0.44	0.16	5.22
29-35	DAVD (min/visit)	2.03	0.46	0.13	6.31
36-42	DAVD (min/visit)	1.99	0.37	0.21	5.35
22-28	FCR (g:g)	2.52	0.45	1.69	4.34
29-35	FCR (g:g)	2.70	0.42	1.75	4.56
36-42	FCR (g:g)	2.80	0.44	1.88	4.73

BW: body weight; DNVF: daily number of visited feeders; DFI: daily feed intake; DTSF: daily time spent at the feeders; DAVD: daily average visit duration; FCR: feed conversion ratio.

With respect to feeding behavior, week-based descriptive statistics indicated that the mean DNVF increased with age, from 15.4 visits/day at 22–28 d to 18.2 visits/day at 36–42 d. A similar trend was observed for the mean DTSF, which increased from 32.7 min/day to 36.3 min/day across the same periods. The mean DAVD remained relatively stable, ranging from 1.99 to 2.12 min/visit.

The mean DFI increased with age, and its standard deviation expanded across successive age stages, indicating that individual differences in feed intake became more pronounced during the later growth period. The mean FCR increased from 2.52 to 2.80, suggesting a slight decline in feed utilization efficiency in the later stage.

### Model performance evaluation

Table 3 summarizes the predictive performance of 16 DFI prediction models developed by combining four ML algorithms (LASSO, RF, GBR, and XGB) with four feature sets (IF, FB, Age_Sex_FB, and IF_FB) across three age windows (22–28 d, 29–35 d, and 36–42 d). Overall, ensemble learning models (RF, GBR, and XGB) consistently outperformed the linear model (LASSO). In addition, the integrated feature set (IF_FB) achieved higher goodness-of-fit and lower prediction errors in most stages, suggesting that the relationships between DFI and the input variables are partly nonlinear and that integrating growth and feeding-behavior information can improve prediction accuracy.

**Table 3:** Performance of machine learning models for predicting daily feed intake of *Wanxi White geese*.

Model	Age range (d)								
	22-28			29-35			36-42		
	MAE	RMSE	R²	MAE	RMSE	R²	MAE	RMSE	R²
GBR-AS_FB	11.57	14.05	0.62	15.21	18.42	0.67	17.61	20.37	0.67
GBR-FB	13.95	15.23	0.59	16.14	21.38	0.65	19.67	24.77	0.63
GBR-IF	14.53	19.15	0.42	18.42	25.12	0.57	23.05	27.04	0.55
GBR-IF_FB	11.81	14.79	0.69	10.93	14.65	0.77	12.33	15.62	0.71
LASSO-AS_FB	11.23	16.41	0.63	16.53	21.80	0.66	21.67	27.21	0.59
LASSO-FB	14.78	17.12	0.58	20.03	24.75	0.57	23.99	27.96	0.52
LASSO-IF	15.65	19.21	0.38	22.21	26.21	0.34	22.51	28.23	0.41
LASSO-IF_FB	13.59	16.45	0.62	20.26	23.23	0.66	21.67	27.21	0.68
RF-AS_FB	11.26	14.43	0.65	16.42	21.78	0.56	14.85	17.03	0.71
RF-FB	10.28	14.96	0.61	16.63	20.87	0.62	14.57	20.23	0.59
RF-IF	12.75	15.61	0.48	20.04	24.76	0.44	23.58	27.05	0.48
RF-IF_FB	10.01	13.96	0.75	11.43	15.10	0.66	14.52	16.37	0.72
XGB-AS_FB	12.39	15.42	0.57	16.22	21.33	0.55	21.41	26.32	0.65
XGB-FB	12.98	15.70	0.64	11.92	15.84	0.62	16.39	20.18	0.71
XGB-IF	12.21	17.11	0.48	18.23	23.63	0.48	19.24	28.21	0.52
XGB-IF_FB	12.73	15.38	0.69	11.02	15.23	0.73	11.27	14.82	0.77

DFI: daily feed intake; GBR: Gradient Boosting Regression; RF: Random Rorest; XGB: eXtreme Gradient Boosting; LASSO: Least Absolute Shrinkage and Selection Operator; IF: individual-level features; FB: feeding behavior features; AS_FB: age, sex, and feeding behavior features; IF_FB: combined individual-level and feeding behavior features.

When comparing feature sets, models using only individual growth features (IF) showed relatively weaker performance. For example, the GBR-IF model achieved an R² of 0.42 during 22–28 d, whereas incorporating feeding behavior features improved the performance of GBR-FB to 0.59, with substantially reduced errors (MAE = 13.95 g, RMSE = 15.23 g). This result indicates that feeding behavior features contribute more directly to the prediction of DFI. The Age_Sex_FB feature set generally performed between FB and IF_FB, implying that when BW information is unavailable, sex and age can partially enhance the explanatory power of behavioral features.

Across all stages, the IF_FB feature set delivered the most robust overall performance. Specifically, during 22–28 d, RF-IF_FB achieved the highest accuracy (R² = 0.75, RMSE = 13.96 g); during 29–35 d, GBR-IF_FB reached the highest R² (0.77); and during 36–42 d, XGB-IF_FB performed best (R² = 0.77, RMSE = 14.82 g). The optimal algorithm differed across age windows, suggesting that the relationship between DFI and behavioral features may vary with age. Taken together, RF/GBR/XGB models with the IF_FB feature set provide more stable and accurate predictions, and thus represent promising candidates for subsequent derivation of FCR from predicted DFI and for growth performance grading. The final hyperparameter settings of these best-performing IF_FB models are provided in Table 4.

**Table 4:** Optimal hyperparameter settings of the best-performing IF_FB models.

Age range (d)	Best-performing model	Final hyperparameter configuration
22-28	RF-IF_FB	n_estimators = 500
		max_depth = 10
		min_samples_leaf = 1
29-35	GBR-IF_FB	n_estimators = 500
		max_depth = 3
		learning_rate = 0.05
36-42	XGB-IF_FB	max_depth = 4
		learning_rate = 0.05
		subsample = 0.8
		colsample_bytree = 0.8

### Feature importance analysis

**Fig. 5:**
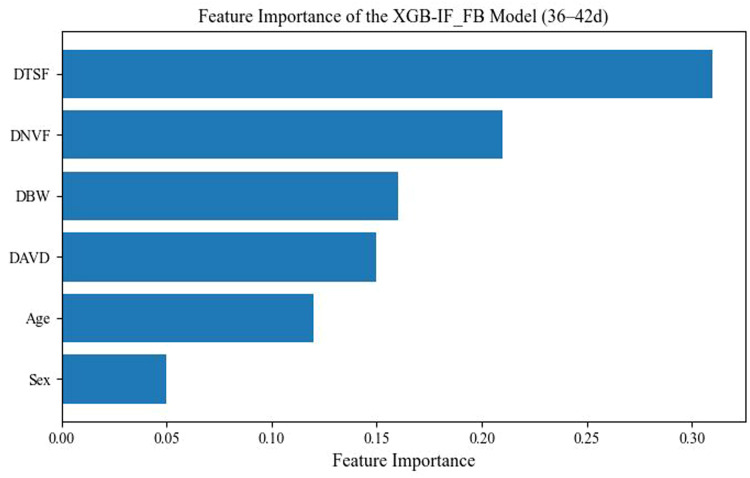
Feature importance of the XGB-IF_FB model for predicting daily feed intake during 36–42 d. DBW: daily body weight; DNVF: daily number of visited feeders; DAVD: daily average visit duration; DTSF: daily time spent at the feeders; XGB: eXtreme Gradient Boosting; IF_FB: combined individual-level and feeding behavior features.

To interpret the prediction mechanism of the model for DFI, feature importance analysis was conducted using the XGB-IF_FB model during the 36–42 d period as an example (Fig. 5). The results showed that DTSF had the highest relative importance (0.31), followed by DNVF (0.21), DBW (0.16), and DAVD (0.15), whereas age (0.12) and sex (0.05) contributed relatively less. These findings indicate that feeding behavior–related variables are key determinants for predicting DFI, suggesting that the time spent at the feeder and visit frequency provide direct signals of feed intake. In addition, DBW offers complementary information regarding the animal’s growth status. In contrast, age and sex exhibited limited marginal explanatory power within a short age window, implying that their effects may be mainly manifested through interactions with growth and behavioral features.

### Results of FCR grading and prediction consistency evaluation

After excluding a small number of individuals with mortality, RFID leg band loss, and other abnormal records, the predicted DFI values generated by the optimal model for each period were used to calculate the predicted FCR. Meanwhile, the observed FCR was derived from the DFI measured by the feeding system. Geese were then classified into growth performance grades based on the mean and standard deviation of the predicted and observed FCR values, respectively. A confusion matrix was constructed to evaluate the agreement between the predicted grades and the observed grades. The grading results and Cohen’s kappa coefficients for different growth periods are presented in Table 5.

**Table 5:** Number of *Wanxi White geese* classified into each predicted and measured FCR efficiency grade during the growing period.

Age range (d)	Measured grade / Predicted grade	Measured low	Measured medium	Measured high	Total	Kappa
22–28 d	Predicted low	26	8	1	35	0.8
	Predicted medium	2	127	1	130	
	Predicted high	0	6	24	30	
	Total	28	141	26	195	
29–35 d	Predicted low	23	7	0	30	0.78
	Predicted medium	1	130	2	133	
	Predicted high	1	8	20	29	
	Total	25	145	22	192	
36–42 d	Predicted low	23	6	0	29	0.77
	Predicted medium	2	127	2	131	
	Predicted high	1	8	21	30	
	Total	26	141	23	190	
22–42 d	Predicted low	19	9	0	28	0.74
	Predicted medium	1	132	1	134	
	Predicted high	1	8	18	27	
	Total	21	149	19	189	

Across the three growth stages (22–28 d, 29–35 d, and 36–42 d), the predicted grades showed high agreement with the observed grades, with kappa coefficients of 0.80, 0.78, and 0.77, respectively, indicating substantial to almost perfect agreement. These results demonstrate that the FCR derived from predicted DFI can accurately identify individuals with different levels of feed efficiency. In particular, the method effectively discriminated between low-FCR (high-efficiency) and high-FCR (low-efficiency) individuals, with few misclassifications across distant grades.

When summarized over the three-week period (22–42 d), the agreement between predicted and observed grades remained high (kappa = 0.74), suggesting that this approach maintains good stability and practical utility over a longer time scale. Overall, most individuals were correctly assigned to the corresponding FCR grade. The medium-FCR group achieved the highest classification accuracy, while individuals with high and low feed efficiency were also effectively distinguished.

## Discussion

This study collected BW and feeder visiting behavior data of geese under floor-rearing conditions using RFID leg bands and electronic feeders, compared the predictive ability of different ML models and feature set combinations for DFI, and further validated the feasibility of efficiency grading based on FCR derived from predicted DFI, thereby providing a basis for low-cost deployment and feed efficiency evaluation in future floor-rearing production scenarios without weighing modules. Overall, the results showed that ensemble learning models (RF, GBR, and XGB) outperformed the linear model (LASSO) in most stages, indicating that the relationships between DFI and the input variables were partly nonlinear, which is consistent with the advantages of ML in handling complex behavioral data (Kyriazos and Poga, 2024; Haseeb et al., 2025).

Across the three time windows, RF achieved the best predictive performance in the 22–28 d stage (RF–IF_FB, R² = 0.75), which is consistent with the results of Tırınk et al. (2023) showing that RF exhibited good robustness in BW prediction during early growth in sheep (R² = 0.735). In the 29–35 d stage, GBR performed best with the integrated feature set (GBR–IF_FB, R² = 0.77), which may be related to a relatively stable relationship between feeding behavior and DFI and a more consistent residual structure in this period, thereby favoring boosting methods that iteratively fit errors, consistent with the findings of Peng et al. (2024) in a study on piglet BW prediction in sows (R² = 0.78). In the 36–42 d stage, XGB achieved the best predictive performance with the integrated feature set (XGB–IF_FB, R² = 0.77), suggesting that the nonlinear relationship between feeding behavior and DFI further increased in the late growth stage and that the advantages of XGB in capturing complex feature interactions became more evident, which is consistent with the conclusion of Önder et al. (2025) that XGB has stronger nonlinear fitting ability in BW prediction of meat-type hybrid does (R² = 0.991).

From the comparison of feature sets, models using only individual growth features showed relatively limited predictive ability, whereas model accuracy improved markedly after incorporating feeding behavior features, indicating that feeder visiting behavior can more directly characterize the individual feeding process. Feature importance analysis showed that DTSF and DNVF had high relative importance. Combined with video surveillance observations, under normal feed availability, the primary behavior of geese after entering the feeders was feeding, and their residence time was highly consistent with the actual feeding time. This phenomenon may be jointly attributed to the need to drink water promptly after consuming dry feed, the limited space inside the feeders, and competition for access within the flock, which together suppress non-feeding residence behaviors and make feeder visiting behavior more specifically reflect the feeding process itself. From physiological and behavioral perspectives, feeding time and visit frequency are closely associated with individual energy demands and feeding motivation, and thus can serve as effective proxy indicators for DFI. Previous studies have reported associations between eating time and intake level in dairy cows and suggested that eating time can be used to infer individual differences in feed intake (Gill et al., 1966; Mol et al., 2016). In addition, the optimal algorithm–feature set combinations differed across weekly age stages, suggesting that the behavior–intake relationship may change dynamically with growth stage and external conditions, which is consistent with reported patterns of changes in feeding rhythms and metabolic demands in birds (Gloutney et al., 2001; Lameris et al., 2021).

From a management perspective, feed efficiency is considered an important economic trait affecting production costs and breeding improvement. Achieving continuous evaluation without additional labor-intensive measurements would facilitate optimized group management and selection decisions (Shirzadifar et al., 2025; Yang et al., 2025). In this study, weekly FCR was calculated based on predicted DFI, and efficiency grading was performed using the mean and standard deviation of FCR. The results showed high agreement between predicted grades and measured grades (kappa > 0.7). These findings indicate that the prediction models can not only estimate DFI with good accuracy but also support the identification of individuals with different levels of feed efficiency for practical production management. Furthermore, the grading results can provide a basis for differentiated decisions across efficiency classes: individuals with low FCR (high efficiency) may be prioritized as candidates for retention or breeding to improve genetic gain; individuals with medium efficiency can be managed as the standard production group and their efficiency may be improved through optimized feeding strategies and health interventions to facilitate upward transitions; and individuals with high FCR (low efficiency) may be targeted for focused interventions (e.g., diet adjustment and health checks) or considered for culling, thereby reducing feed wastage and optimizing flock structure.

This study still has several limitations. First, the models were developed under a single diet condition, with the aim of controlling the confounding effects of dietary composition on DFI and thereby more clearly evaluating the effects of individual growth traits and feeding behavior on DFI. However, because DFI and related behaviors are sensitive to diet type and nutrient level, the generalizability of the proposed models under different dietary conditions remains to be further validated. Previous studies have shown that feed cost or formulation differences can alter feeding behavior, FI, and BW gain in geese (Arroyo et al., 2013), and that different diet types may also affect growth performance and meat quality in Yangzhou geese (Song et al., 2017). Second, environmental factors were not included in the present study. Temperature and lighting have been demonstrated to influence feeding behavior and FI in poultry, which may lead to potential bias when the model is applied across different seasons or housing conditions (Diarra and Tabuaciri, 2014; Rodrigues and Choct, 2019). In addition, model performance was evaluated using data collected over a continuous three-week period (22–42 d of age), and potential changes in the relationship between feeding behavior and DFI over longer growth periods were not addressed. Previous studies have modeled and predicted animal feed intake over longer time scales, highlighting the importance of extended observation windows (Martin et al., 2021; Taylor and Kyriazakis, 2021).

In summary, this study demonstrated the feasibility of predicting DFI of geese under floor-rearing conditions using BW and feeder visiting behavior features, and further verified the application potential of deriving FCR from predicted DFI for efficiency grading. Future integration of multiple diet conditions, environmental variables, and longer-term datasets is expected to further improve model generalization and practical deployability, thereby providing technical support for precision feeding and individual efficiency selection in the goose industry.

## Conclusion

Under floor-rearing conditions, this study collected BW and feeder visiting behavior data of geese using RFID leg bands and an electronic feeder system, developed DFI prediction models, and further verified their applicability for feed efficiency evaluation. The results showed that ensemble learning models generally outperformed the linear model, and that the integrated feature set combining individual growth traits and feeding behavior information exhibited more stable performance, indicating pronounced nonlinear relationships between DFI and the input variables and demonstrating that multi-source information fusion can effectively improve prediction accuracy. Stage-wise evaluation revealed that the optimal models differed across weekly age periods, suggesting that the relationship between feeding behavior and DFI may change dynamically with growth stage. Feature importance analysis further indicated that behavioral indicators such as time spent at the feeders and visit frequency contributed more to DFI prediction and can serve as key proxy features for characterizing the feeding process.

From a production management perspective, estimating weekly FCR based on predicted DFI and conducting efficiency grading showed good consistency, enabling reliable discrimination of individuals with different levels of feed utilization efficiency. These findings provide methodological support and technical evidence for feed efficiency screening, group management, and precision feeding decision-making of geese under floor-rearing conditions.

## Care and use of animal

All experimental protocols complied with the requirements of the Animal Ethics Committee of South China Agricultural University (SCAU). The sensors used in this experiment were deployed by professional farm staff, with no additional handling or contact with the animals. The animals were managed in accordance with the farm's standard practices, avoiding any unnecessary discomfort.

## Declaration of generative AI and AI-assisted technologies in the manuscript preparation process

During the preparation of this work the authors used ChatGPT to improve readability and language (e.g., reformulation of sentences). After using this tool/service, the authors reviewed and edited the content as needed and take full responsibility for the content of the publication.

## CRediT authorship contribution statement

**Deqin Xiao:** Funding acquisition. **Weichao Lin:** Writing – original draft. **Youfu Liu:** Writing – review & editing. **Shaohai Peng:** Supervision. **Dakang Guo:** Data curation. **Kejian Liu:** Project administration.

## Disclosures

The authors declare that they have no known competing financial interests or personal relationships that could have appeared to influence the work reported in this paper.
